# Insights From Twitter Conversations on Lupus and Reproductive Health: Protocol for a Content Analysis

**DOI:** 10.2196/15623

**Published:** 2020-08-26

**Authors:** Oleg Stens, Michael H Weisman, Julia Simard, Katja Reuter

**Affiliations:** 1 Department of Internal Medicine Harbor-UCLA Medical Center Torrance, CA United States; 2 David Geffen School of Medicine at UCLA Los Angeles, CA United States; 3 Division of Epidemiology Department of Health Research and Policy Stanford University Palo Alto, CA United States; 4 Institute for Health Promotion and Disease Prevention Research Department of Preventive Medicine Keck School of Medicine of USC Los Angeles, CA United States; 5 Southern California Clinical and Translational Science Institute Keck School of Medicine of USC University of Southern California Los Angeles, CA United States

**Keywords:** fertility, infodemiology, infoveillance, listening, lupus, monitoring, patient opinion, reproductive health, surveillance, Twitter, social media, social network

## Abstract

**Background:**

Systemic lupus erythematosus (SLE) is the most common form of lupus. It is a chronic autoimmune disease that predominantly affects women of reproductive age, impacting contraception, fertility, and pregnancy. Although clinic-based studies have contributed to an increased understanding of reproductive health care needs of patients with SLE, misinformation abounds and perspectives on reproductive health issues among patients with lupus remain poorly understood. Social networks such as Twitter may serve as a data source for exploring how lupus patients communicate about their health issues, thus adding a dimension to enrich our understanding of communication regarding reproductive health in this unique patient population.

**Objective:**

The objective of this study is to conduct a content analysis of Twitter data published by users in English in the United States from September 1, 2017, to October 31, 2018, in order to examine people’s perspectives on reproductive health among patients with lupus.

**Methods:**

This study will analyze user-generated posts that include keywords related to lupus and reproductive health from Twitter. To access public Twitter user data, we will use Symplur Signals, a health care social media analytics platform. Text classifiers will be used to identify topics in posts. Posts will be classified manually into the a priori and emergent categories. Based on the information available in a user’s Twitter profile (ie, username, description, and profile image), we will further attempt to characterize the user who generated the post. We will use descriptive statistics to analyze the data and identify the most prevalent topics in the Twitter content among patients with lupus.

**Results:**

This study has been funded by the National Center for Advancing Translational Science (NCATS) through their Clinical and Translational Science Awards program. The Institutional Review Board at the University of Southern California approved the study (HS-18-00912). Data extraction and cleaning are complete. We obtained 47,715 Twitter posts containing terms related to “lupus” from users in the United States, published in English between September 1, 2017, and October 31, 2018. We will include 40,885 posts in the analysis, which will be completed in fall 2020. This study was supported by funds from the has been funded by the National Center for Advancing Translational Science (NCATS) through their Clinical and Translational Science Awards program.

**Conclusions:**

The findings from this study will provide pilot data on the use of Twitter among patients with lupus. Our findings will shed light on whether Twitter is a promising data source for learning about reproductive health issues expressed among patients with lupus. The data will also help to determine whether Twitter can serve as a potential outreach platform for raising awareness of lupus and reproductive health and for implementing relevant health interventions.

**International Registered Report Identifier (IRRID):**

DERR1-10.2196/15623

## Introduction

### Background and Rationale

Lupus is a chronic autoimmune disease that can affect any part of the body (skin, joints, or vital organs) [1,2]. Estimates from recent population-based studies in the United States report the prevalence of systemic lupus erythematosus (SLE), the most common form of lupus, to be between 60 and 80 per 100,000, although this prevalence varies greatly by age, gender, race, and ethnicity. It is generally accepted that SLE is much more prevalent in women than men (up to 9 times higher prevalence) and that people of color have both higher prevalence rates and more severe manifestations of the disease compared to White populations. Rates as high as 196 per 100,000 have been reported in African American women [3,4].

SLE predominantly impacts women during the childbearing years, affecting contraception, fertility, and pregnancy, which are matters of importance to the patients and their family members. Providing care to pregnant patients with lupus is an important challenge for their families and the health care system. Although quite a few studies in the modern era have clarified the field of reproductive health care for SLE patients [5], misinformation abounds. Perspectives on reproductive health issues, especially those regarding medication risks and benefits, among patients with lupus and their family members remain poorly understood. In this study, we define the term “perspective” as an expression of thought, viewpoint, and attitude toward the reproductive health issues that have been identified in the literature, such as pregnancy prevention, pregnancy termination, pregnancy planning, conception, and concerns and management of childbirth [6]. A better understanding of the perspectives on reproductive health issues among patients with lupus can inform and improve the advocacy and education efforts to address the gaps in care, dispel misconceptions, and more effectively assist patients in making family planning decisions.

### Social Media

Social media consists of web-based and mobile technologies that allow users to view, create, and share information online and participate in social networking [7-9]. Social media provides a unique source for data mining of health conditions and concerns, serving as a massive focus group [10-12]. A total of 72% of American adults use at least some type of social media [13], which provides an unprecedented opportunity for delivering information to reach large segments of the population [14] as well as hard-to-reach subpopulations [15,16]. Data from social networks such as Twitter, Instagram, and YouTube that allow users to discuss topics of their choice “unprimed by a researcher and without instrument bias” [10] can be used to capture and describe the social and environmental context in which individuals experience and describe their health conditions and concerns [17].

### Twitter

Based on Pew Research data from 2019, nearly a quarter (22%) of adults in the United States use the social network Twitter; 40% of those are daily users [13]. Twitter allows users to post “tweets”, short posts that are limited to 280 characters [18]. Users can search for any public tweet and engage with it through “like,” “reply,” and “retweet” (repost). Twitter is primarily public. Basic account information such as profile username, description, and location remains public. However, users can choose to keep their tweets protected to make them private or visible to subsets of users such as their followers or those they decided to follow [19,20]. Due to the more public nature of Twitter, previous research suggested that Twitter provides a “rich and promising avenue for exploring how patients conceptualize and communicate about their specific health issues” [21]. The increasing use of Twitter among the members of communities with disease is further evidenced by the abundance of disease-specific and health-related hashtags used in the tweets [22-24]. A hashtag is a word or phrase preceded by a hash or pound sign (#), which is used to identify tweets on a specific topic (eg, #lupus, #spoonies). These hashtags are used by users to assign their tweets to a topic and join ongoing conversations. Users can click on a hashtag and view all of the tweets that include the same hashtag; hence, discuss the same topic. This allows users to form online communities and share their health concerns, disease experience, and questions with other users [25]. However, there is little information about the use of social media among patients with lupus.

### Previous Research on Social Media and Lupus

The emergence of social media has created new sources of analyzable data [12] and led to new research fields, such as infodemiology and infoveillance [11]. The data social media users generate through their online activities is referred to as their digital footprint [26] or social mediome [27].

Previous research examined user-generated content about lupus on Facebook [28]. Hale et al [28] looked at the representation of health conditions and found that lupus-related pages ranked the highest for patient support. Additionally, a patient commentary highlighted social media use (Twitter, in particular) by patients with lupus to find rheumatologists, specialist care, and peers and to build awareness of their health needs and experiences [29]. Health surveillance researchers have used Twitter data to gain insights into the public perspectives on a variety of diseases and health topics such as influenza, autism, schizophrenia, smoking, and HIV/AIDS [30-35]. In some cases, social media user data demonstrated a correlation between the disease prevalence and frequency with which Twitter users discussed that disease [36]. To our knowledge, there are no studies that have leveraged Twitter to gain a better understanding of the perspectives of patients with lupus on reproductive health issues.

### Study Objective and Research Questions

The objective of this study is to conduct a content analysis of tweets published in English by users in the United States during the period from September 1, 2017, to October 31, 2018, and to examine the perspectives of patients with lupus on reproductive health issues. We intend to answer the following research questions outlined in Textbox 1.

Our findings will shed light on whether Twitter is a promising data source for garnering insights about reproductive health concerns among the patients with lupus. The data will also help determine whether Twitter can serve as a potential outreach platform for raising awareness of lupus and reproductive health and for implementing relevant health interventions.

**Research questions.**

- What is the volume of Twitter users who talk about lupus and reproductive health issues such as pregnancy prevention; pregnancy termination; and planning, conception, and management of pregnancy?
- How many of these users are patients with lupus?
- What are the perspectives, issues, and concerns that the patients with lupus express regarding their reproductive health?
- What are the demographics (ie, gender, race/ethnicity) of these patients with lupus on Twitter?

## Methods

### Data Collection

This qualitative study will analyze user-generated posts that include keywords related to lupus and fertility from the social network Twitter.

### Data Source

To access public Twitter user data, we used Symplur Signals [37], a health care social media analytics company that maintains the largest publicly available database of health care– and disease-related conversations with the globally recognized Healthcare Hashtag Project. Symplur Signals extracts data from the Twitter representational state transfer (REST) application programming interface (API) and makes those available to researchers; those data are commonly used in peer-reviewed research [22,23,38-41]. We extracted data from Twitter using Symplur Signals user interface, searching for the relevant keywords and hashtags (Multimedia Appendix 1) from September 1, 2017, to October, 31, 2018. The data were provided in a spreadsheet, which we analyzed on local computers.

### Search Filters

We utilized the framework suggested by Kim et al [42] for data collection, quality assessment, and reporting of standards. Twitter posts containing lupus-related terms were obtained for the period ranging from September 1, 2017, to October 31, 2018. The list of terms we used to collect the sample of tweets is shown in Multimedia Appendix 1. These terms can appear in the post or in an accompanying hashtag, for example, lupus or #LupusChat. LupusChat is a global health organization based in New York City, founded in 2012 by Tiffany Marie Peterson, a patient advocate who was diagnosed with SLE. The biweekly Twitter chat hosted by LupusChat is popular among patients with lupus to discuss related health concerns and the impact lupus has on their lives [43]. The selected keyword and hashtags are based on expert knowledge from clinicians and social media experts as well as on a systematic search of topic-related language using the Symplur Signals database. For each term, we viewed about 50 tweets to determine inactive as well as new keywords and hashtags that were being used in the lupus-related posts, particularly by patients. We will analyze the tweets from the patients with lupus to identify the issues and concerns they express regarding their reproductive health. Previous research has identified multiple challenges experienced by patients with SLE, for example, fertility preservation, optimal care during pregnancies, risks of adverse maternal or fetal outcomes, safety of contraceptive methods for women, and effects of dermatologic medications on male fertility [44-47].

### Data Cleaning

The following types of posts were excluded: (1) non-English language tweets (which were identified using the methodology by Lui and Baldwin [48] and the language detection API of detectlanguage.com), (2) retweets that were originally composed/posted by other users, and (3) tweets that originated from outside the United States. We did not include retweets in the analysis dataset, as we intend to examine the patients’ original perspectives on reproductive health issues. The locations of the users were determined using a mapped location filter as defined using “Profile Geo 2.0” algorithm (Gnip Inc) [49]. The algorithm uses a number of data points to determine a user’s location, including the self-reported “Location” in the user profile and geotracking data, if available.

Furthermore, we relied on machine learning to recognize tweets by social bots or marketing-oriented accounts that could possibly influence the results and introduce bias [50,51]. Automated accounts on Twitter created by industry groups and private companies contribute to the corpus of Twitter data to influence discussions and promote specific ideas or products [28]. To identify those bias accounts, we identified a user account responsible for each tweet collected in the dataset and analyzed its recent history, interactions, and metadata to determine the account was a social bot, a computer algorithm designed to automatically produce content and engage with humans on Twitter [50]. Tweets from these accounts “pollute social and health research data sets” [52]. They were identified and excluded from the dataset of tweets from patients with lupus. Bot accounts were identified using a system that analyzes the account’s network (diffusion patterns), user (metadata), friends (account’s contacts), temporal pattern (tweet rate), and sentiment (content of message), as previously described. The system detects bots with a 95% success rate [50].

### Data Analysis

#### Coding

Two independent team members will be responsible for coding based on a set of a priori classifiers listed in Multimedia Appendices 2 and 3. We will use the profile information (ie, username, description, and profile image) of a Twitter account, which generated a relevant post, to characterize its user and determine if that user is a patient with lupus (Multimedia Appendix 3). Specifically, we will check if these users self-identify as patients with lupus in their profile description.

We will then code the tweets from patients with lupus (Multimedia Appendix 2). A tweet will be classified as the one by a patient with lupus—if that user has already been identified as such through examination of their Twitter profile or if the tweet describes lupus symptoms or lupus-related events in the first person (eg, My doctor had to change my medications today to the ones that are safe in pregnancy).

Additionally, we will code the person’s gender and race/ethnicity if the profile contains sufficient information to do so. Cohen’s kappa will be calculated for each code category to assess interrater reliability [53,54]. Once we establish concordance in the coder’s classification with κ>0.8 for each coding category, the remaining data will be divided between the 2 coders. Principal investigators of the project will help establish consensus in instances where coders disagree.

#### Statistical Analysis

The analysis will rely on public, anonymized data and will adhere to the terms and conditions, terms of use, and privacy policies of Twitter. This study will be conducted under the approval from the institutional review board of the authors’ university. No tweets will be reported verbatim in the findings to protect the privacy of the users. Representative examples of tweets within each category will be selected to illustrate additional themes and will be shown as paraphrased quotes.

We will use descriptive statistics to identify the most prevalent topics in the Twitter content. Units of analysis will be unique terms in tweets, number of tweets, and number of users with lupus. For each analysis, we will present the findings in a confusion matrix, where diagonal lines would indicate the prevalence of a topic and off-diagonal lines, a topic overlap. The number of posts containing 2 or more topics would be found at the intersection of the matrix for these topics. We will further describe the patient characteristics focusing on gender and race/ethnicity, as reported on Twitter.

### Data Privacy and Confidentiality

Study data will be stored using the Research Electronic Data Capture (REDCap) system at the University of Southern California (USC). REDCap is a secure, web-based application designed to support data capture for research studies [55]. It provides (1) an intuitive interface for validated data entry, (2) audit trails for tracking data manipulation and export procedures, (3) automated export procedures for seamless data downloads to common statistical packages, and (4) procedures for importing data from external sources. This database system facilitates the required provision of data to the USC Institutional Review Board, National Institutes of Health (NIH), and Food and Drug Administration (FDA).

Usernames will be initially available to the coders when they are examining the profiles to record the user demographics and determine whether a user is a patient with lupus. Profile usernames will then be redacted from the data file and replaced with unique numeric code identifiers before coders start examining the tweets. The link between the unique codes and the identifiable elements will be kept in a separate file. Thus, the coders will not be able to simultaneously view the identifiable elements of a Twitter profile and tweets made by that Twitter user. Additionally, any identifying and personal health information that the coders might find in the dataset of the tweets will be redacted by the coders. We will retain the data only for use in this project and destroy the identifiable information (tweet ID, tweet URL, thumbnail/URL of profile picture, username, and display name) prior to the data analysis. Given the sensitive nature of the topic “lupus and fertility,” this step will be taken to protect the privacy of pregnant women whose tweets might be included in the data sample.

### Risk Analysis

This research has minimal risk. We will use publicly available data from the social network Twitter. Identifiable information such as human subjects’ names and Twitter usernames will not be included in the analysis dataset. We will further abide by the USC Institutional Review Board regulations and the USC Privacy of Personal Information policy. All data will be entered into a password-protected computer database. The data will be stored using appropriate secure computer software and encrypted computers.

### Dissemination of Study Findings

The authors plan to publish the study findings in a peer-reviewed journal and present those at relevant conferences (to be determined at a later date). All the listed authors and contributors comply with the guidelines of the International Committee of Medical Journal Editors on author inclusion in a published work.

## Results

Study approval was obtained from the Institutional Review Board at USC (Protocol HS-18-00912) (Multimedia Appendix 4). Data extraction and cleaning are complete. We obtained 47,715 tweets containing terms related to “lupus” from users in the United states that were posted in English during the period September 1, 2017, to October 31, 2018. We will include 40,885 posts in the analysis. The detailed data extraction and cleaning flowchart is included in Multimedia Appendix 5. Data analysis will be completed in fall 2020.

## Discussion

### Limitations

This exploratory pilot study is limited to Twitter conversations from the patients of lupus who use the words lupus and SLE or the related hashtags in their tweets. As a result, tweets that share lupus-related experiences of patients without using the related terms and hashtags will be excluded from the study.

We recognize that this social media research and intervention favor those with the internet access and that this limitation could lead to potential bias in the research data. The generalizability of this study is also somewhat limited because the study excludes tweets from outside of the United States and tweets written in languages other than English. However, social media users “have grown more representative of the broader population.” Twitter is used by 24% of Black Americans, 21% of White Americans, and 25% of Hispanic Americans. Twitter use is more common among younger (38% use among persons aged 18 to 29 years vs 7% use among those older than 65 years); educated (32% among college graduates vs 13% among those with a high school diploma or less); and urban (26% urban users vs 13% rural users) demographic [13].

### Practical Significance

This pilot project will provide preliminary data and an insight into the application of publicly available Twitter data to gain a better understanding of the patients with lupus and their perspectives on reproductive health issues. If successful, our findings will shed light on whether Twitter provides a promising data source for garnering perspectives on reproductive health issues expressed by the patients with lupus. The data will also help to determine whether Twitter can be a potential outreach platform for raising awareness of lupus and reproductive health and for implementing the related health interventions.

## Appendix

Multimedia Appendix 1 Keywords and hashtags used for the Twitter search to assess Twitter conversations about lupus and reproductive health.

Multimedia Appendix 2 Code categories to identify main themes in Twitter posts about lupus and reproductive health.

Multimedia Appendix 3 Code categories to classify Twitter users.

Multimedia Appendix 4 IRB approval notice.

Multimedia Appendix 5 Data extraction and cleaning flow diagram.

## Abbreviations

API: Application Programming Interface
FDA: Food and Drug Administration
NCATS: National Center for Advancing Translational Science
NIH: National Institutes of Health
REDCap: Research Electronic Data Capture
REST: Representational State Transfer
SLE: systemic lupus erythematosus
USC: University of Southern California
