# Supplementary material for: Insights From Twitter Conversations on Lupus and Reproductive Health: Protocol for a Content Analysis
Source: JMIR Res Protoc. 2020 Aug 26;9(8):e15623. doi: 10.2196/15623 (PMC7481870; doi:10.2196/15623)
Supplement: Multimedia Appendix 1 [file resprot_v9i8e15623_app1.pdf]

Multimedia Appendix 1. Keywords and hashtags used for the Twitter search to assess Twitter conversations about lupus and reproductive health.

Topic	Keywords	Hashtags
**Lupus**	Lupus	#lupus<br>#lupuschat<br>#lupusstyle<br>#WorldLupusDay<br>#TalkToMeAboutLupus<br>#LupusFeelsLike<br>#LupusTaughtMe<br>#SLE<br><br>**Exclude**:<br>Spanish and other languages: #JornadaLupus, #LupusTGN<br>Non-active:<br>#LupusPFDD, #NIAMSLupusChat<br>#lupuschat<br>#lupusstyle<br>#lupusawareness<br>#WorldLupusDay<br>#TalkToMeAboutLupus<br>#LupusFeelsLike<br>#LupusTaughtMe<br>#LupusAwareness<br>#lupusadvocacy<br>#lupusadvocate<br>#strongerthanlupus<br>#lupussurvivors<br>#lupuswarrior<br>#sle<br>#LupusResearchMatters<br>#AdvocateForLupus<br>#LupusAwarenessNYS<br><br>**Exclude**:<br>Spanish and other languages: #JornadaLupus, #LupusTGN<br>Non-active:<br>#LupusPFDD, #NIAMSLupusChat
