# Supplementary material for: Insights From Twitter Conversations on Lupus and Reproductive Health: Protocol for a Content Analysis
Source: JMIR Res Protoc. 2020 Aug 26;9(8):e15623. doi: 10.2196/15623 (PMC7481870; doi:10.2196/15623)
Supplement: Multimedia Appendix 2 [file resprot_v9i8e15623_app2.pdf]

Multimedia Appendix 2. Code categories to identify main themes in Twitter posts about lupus and reproductive health.

Code category	A priori codes/variables and definitions (data dictionary code value)	Emergent codes/variables and definitions (code value)
Pregnancy planning	● Conception (defined as: trying to conceive)<br>● Fertility (defined as: effect of lupus on conception and success of pregnancy, e.g., miscarriage)<br>● Medication (defined as: medication lupus patients should or shouldn't take, stopping medication, safety, compatibility, unsafe medications, teratogenicity, side-effects in patients planning on becoming pregnant)<br>● Indeterminate, or other topics related to pregnancy planning	
Pregnancy management	● Monitoring (defined as: seeing/speaking with doctor, knowing what type of physician to consult)<br>● Flares (defined as: mention of flares and remission in pregnancy)<br>● Risks and complications (defined as: Pre-clampsia, Pre-term birth, Small-for-gestational-age neonates)<br>● Medication (defined as: medication lupus patients should or shouldn't take, stopping medication, safety, compatibility, unsafe medications, teratogenicity, side-effects in patients who are already pregnant)<br>● Indeterminate, or other topics related to pregnancy management	

Delivery/Labor/Birth	• Pre-term birth<br>• Cesarean section<br>• Indeterminate, or other topics related to delivery/labor/birth	
Pregnancy prevention	• Contraception<br>• Planned parenthood<br>• Indeterminate, or other topics related to pregnancy prevention	
Pregnancy termination	• Elective abortion<br>• Spontaneous abortion/miscarriage<br>• Stillbirth<br>• Indeterminate, or other topics related to pregnancy termination	
Male partners of women with lupus	• Birth control options<br>• Family planning<br>• Indeterminate, or other topics related to male partners of women with lupus	
Male SLE patients regarding pregnancy and fatherhood	• Birth control<br>• Fertility<br>• Medication during conception<br>• Adverse fetal outcomes<br>• Passing on lupus<br>• Indeterminate, or other topics related to male SLE patients	
