# Supplementary material for: Insights From Twitter Conversations on Lupus and Reproductive Health: Protocol for a Content Analysis
Source: JMIR Res Protoc. 2020 Aug 26;9(8):e15623. doi: 10.2196/15623 (PMC7481870; doi:10.2196/15623)
Supplement: Multimedia Appendix 3 [file resprot_v9i8e15623_app3.pdf]

Table 3. Code categories to classify Twitter users.

Code category	A priori codes/variables and definitions (data dictionary code value)	Emergent codes/variables and definitions (code value)
**SLE Status**	• Patient with lupus<br>• Partner of patient with lupus<br>• Healthcare professional<br>• Other or could not be determined	
**Sex**	• Female<br>• Male<br>• Transgender<br>• Could not be determined	
**Race**	• White or Caucasian<br>• Person of color<br>• Could not be determined	
