# Supplementary material for: Insights From Twitter Conversations on Lupus and Reproductive Health: Protocol for a Content Analysis
Source: JMIR Res Protoc. 2020 Aug 26;9(8):e15623. doi: 10.2196/15623 (PMC7481870; doi:10.2196/15623)
Supplement: Multimedia Appendix 4 [file resprot_v9i8e15623_app4.pdf]

Proposal #HS-18-00912

University of Southern California Institutional Review Board
1640 Marengo Street, Suite 700
Los Angeles, California 90033-9269
Telephone: (323) 442-0114
Fax: (323) 224-8389
Email: irb@usc.edu

Date: Mar 28, 2019, 10:21am
To: Katja Reuter, PhD
Assistant Professor Of Clinical Preventive Medicine
PREVENTIVE MEDICINE

From: University of Southern California Institutional Review Board

---

TITLE OF PROPOSAL:
Lupus and Reproductive Health: Insights from User Conversations on the Social Network Twitter (Lupus and Reproductive Health: Twitter Study)

Action Date: **3/28/2019** Action Taken: **Approve**
Committee: Institutional Review Board
Note: Your iStar application and attachments were reviewed by the expedited mechanism by Julie Slayton, EdD on March 28, 2019.

The project was APPROVED.

The materials submitted and considered for review of this project included:
1. Revised iStar application dated 3/27/2019
2. Response to contingencies from 3/22/2019 and 3/27/2019
3. Revised Study protocol, dated 3/22/2019
4. Data collection forms

Based on the information submitted for review, this study qualifies for expedited review according to §46.110(b) (5).

In approving this research, the IRB determined that all of the following requirements (45CFR 46.111) were satisfied: (1) Risks to subjects are minimized: (i) by using procedures which are consistent with sound research design and which do not unnecessarily expose subjects to risk, and (ii) whenever appropriate, by using procedures already being performed on the subjects for diagnostic or treatment purposes. (2) Risks to subjects are reasonable in relation to anticipated benefits, if any, to subjects, and the importance of the knowledge that may reasonably be expected to result. In evaluating risks and benefits, only those risks and benefits that may result from the research are considered (as distinguished from risks and benefits of therapies subjects would receive even if not participating in the research). (3) Selection of subjects is equitable (the purposes of the research and the setting in which the research will be conducted were taken into account). (4) Informed consent will be sought from each prospective subject or the subject's legally authorized representative, in accordance with, and to the extent required by 45CFR 46.116. (5) Informed consent will be appropriately documented, in accordance with, and to the extent required by 45CFR 46.117. (6) When appropriate, the research plan makes adequate provision for monitoring the data collected to ensure the safety of subjects. (7) When appropriate, there are adequate provisions to protect the privacy of subjects and to maintain the confidentiality of data.

As the Principal Investigator, you are required to ensure that this research and the actions of all project personnel involved in conducting the study will conform with the research project and its modifications approved by the IRB; HHS regulations (45CFR46); FDA regulations (21CFR50,56); International Conference on Harmonization Good Clinical Practice Consolidated Guideline; IRB Policies and Procedures and applicable state laws. Failure to comply may result in suspension or termination of your research project, notification of appropriate governmental agencies by the IRB, and/or suspension of your freedom to present or publish results. Any proposed changes in the research project must be submitted, reviewed and approved by the IRB before the change can be implemented. The only exception is a change necessary to eliminate apparent immediate hazards to the research subjects. In such a case, the IRB should be promptly informed of the change following its implementation for IRB review. You must inform the IRB immediately if you become aware of any violations of HHS regulations (45CFR46), FDA regulations (21CFR50,56), applicable state laws or IRB Policies and Procedures for the protection of human subjects. You are required to notify the IRB office in the event of any action by the sponsor, funding agency or FDA, including warnings, suspension or termination of your participation in this trial. You must maintain all required research records and recognize the IRB is authorized to inspect these records.

You must inform the IRB of any unanticipated adverse event or injury no later than ten (10) business days following the time it becomes known that a subject suffered an adverse event/injury. To report external or internal adverse events to the IRB, you must complete and

submit the Reportable Event forms in iStar. Furthermore, you must inform the IRB immediately of any significant negative change in the risk/benefit relationship of the research as originally presented in the protocol and approved by the IRB.

** *This project is not subject to requirements for continuing review.*

INFORMED CONSENT

The request for a WAIVER OF INFORMED CONSENT is approved as iStar item 24.5 adequately documents that: (a) the research involves no more than minimal risk to the subjects; (b) the waiver or alteration will not adversely affect the rights and welfare of the subjects; (c) the research could not practicably be carried out without the waiver or alteration; and (d) the subjects will be provided with additional pertinent information after participation (where appropriate).

HIPAA AUTHORIZATION

The HIPAA Privacy Rule will not apply to this research. The investigator certifies that he/she is not accessing, using or obtaining protected (i.e., identifiable) health information held by: a) a health care provider (e.g., physician or other health care practitioner, hospital, clinic, nursing home); b) health plan (e.g., group health plan, insurance company, (HMO); or c) health care clearinghouse (e.g., billing service) that is governed by the HIPAA privacy federal regulations.

Attachments:

Approved Documents: view

This is an auto-generated email. Please do not respond directly to this message using the "reply" address. A response sent in this manner cannot be answered. If you have further questions, please contact your IRB Administrator or IRB/CCI office.

The contents of this email are confidential and intended for the specified recipients only. If you have received this email in error, please notify istar@usc.edu and delete this message.

Print Close

1. Project Identification and Abstract

1.1. * **Type of Submission**:

- (●) **Research Protocol or Study on Human Subjects**
- ( ) Use of Humanitarian Use Device (Not Research)
- ( ) Rely on another IRB (Ceded)

1.2. * **Full Title of Research Protocol**
Lupus and Reproductive Health: Insights from User Conversations on the Social Network Twitter

1.3. * **Short Title**
Lupus and Reproductive Health: Twitter Study

1.4. **Abstract: Provide a simple explanation of the study and briefly address (in 1 to 2 sentences) each of the following points: rationale; intervention; objectives or purpose; study population or sample characteristics; study methodology; description of study arms (if appropriate); study endpoints or outcomes; follow-up; statistics and plans for analysis.**
Rationale: Lupus is a chronic autoimmune disease that can affect any part of the body (skin, joints, and/or vital organs). A better understanding of attitudes towards reproductive health among lupus patients could inform and improve advocacy and education to address gaps in care, dispel misconceptions, and more effectively assist patients in making family planning decisions.

Intervention: N/A

Objectives/Research questions: The objective of this study is to conduct a content analysis of Twitter data published by users (in English) in the U.S. between 9/1/2017 and 10/31/2018 in order to examine attitudes toward reproductive health issues among patients with lupus. Please see the study protocol for the detailed research questions.

Study population: Data from Twitter users in the US

Study methodology: Observational study

Plans for analysis:

Coding
Text classifiers will be used to identify topics in posts. Posts will be classified manually into the a-priori and emergent specific categories. Two independent team members (PI and student/tbd) will review the Twitter messages to code them based on the coding categories. Please note that any identifying and personal health information will be redacted from the dataset by the coders.

Statistical analysis
We will use descriptive statistics to analyze the data and identify the most prevalent topics in the Twitter content. Units of analysis will be unique terms in posts as well as the number of Twitter messages and users. Representative examples of tweets within each category will be selected to illustrate additional themes and will be shown as direct quotes, redacting information that might identify a contributor�s identity.

Additionally, for posts mentioning keywords of interest, the Symplur Signals platform will be used to

create word clouds to summarize the frequencies of co-occurrence mentions, with the size of the text represented as the relative frequency of each term compared with other terms. We will also use the platform to identify the top five languages used to talk about the topic.

1.5. * **Select which IRB you are requesting review from:**
USC-Health Sciences (HSC)

1.6. * **To the investigator's knowledge, does the Institution have financial and/or intellectual property interests in the sponsor or the products used in this project?** *An institutional conflict may occur when a financial interest of the institution has the potential to bias the outcome of research conducted by its employees or students or to create an unacceptable risk to human subjects.*
○ Yes ● **No**

2. Study Personnel

2.1. Study Personnel and their roles:

	Last Name	First Name	Organization	Study Role	Certifications	Obtain Consent	Interact with Participants	Access Identifiable Data
View	Reuter	Katja	PREVENTIVE MEDICINE	Principal Investigator	HS	no	no	no

2.2. Is the Principal Investigator a student, resident, fellow, postdoctoral scholar, other trainee, or visiting scholar at USC/CHLA?
○ Yes ● **No**

2.4. Does this study require Cancer Center Committee (CIC) approval?
○ Yes ● **No**

2.4.1. Are Cancer Patients Involved? ○ Yes ○ No

2.5. Specify the group/organization who has reviewed this study for scientific merit:

○ Federal Agency (e.g. FDA, NIH, CDC, DOE, NSF, DOJ, etc.)

○ USC Norris Clinical Investigations Committee

○ Doctoral Dissertation Committee

○ Other

● **None**

3. Required Approvals (for a study already submitted to the IRB)

This screen indicates the approvals received once the proposal has been submitted.

3.1. Pending Division/Department Approvals:

Name	Division/Department	Parent Campus

There are no items to display

3.2. Received Division/Department Approvals:

Name	Division/Department	Parent Campus
PREVENTIVE MEDICINE	Department	USC-Health Sciences (HSC)

3a.3. Other campus committees, services or departments that need to review and approve this protocol:

Committee Name	Committee Chair	Approval Memo

There are no items to display

3a.4. Will the research be conducted through the CTU?

○ Yes ● **No**

4. Funding Information

4.1. * **What existing, planned, or pending support will be used for this study?** (check all that apply)

- [ ] Cooperative Group (SWOG, COG, RTOG, etc.)
- [x] **CTSI**
- [ ] Department of Defense (DOD) Funds
- [ ] Departmental/Institutional Funds
- [ ] Federal Grant/Contract
- [ ] Foundation Grant/Contract
- [ ] Industry
- [ ] Intramural/Internal Grant
- [ ] Residual Funds
- [ ] State or Local Grant/Contract
- [ ] Subcontract from another institution
- [ ] No Funding

- [ ] Other

4.1.1 Will you be submitting a Just-in-Time (JIT) request?

○ Yes ● **No**

4.2. If the funding source has undergone separate review by the IRB (i.e., cooperative group grants, umbrella grants, multi-project/program grants, center grants), try to select it from the list using the "Add" button. If the funding source is not displayed in the list, enter the information in question 4.4.

Grant #	Principal Investigator	Grant Title
There are no items to display		

4.2.1. If the grants selected in question 4.2 fund multiple studies, please attach the specific pages of the grant that are relevant to THIS study.

Name	Version	Modified
There are no items to display		

4.3. If applicable, select a clinical trial from the TRUE2 system: (Important Note: As of April 1, 2016, the TRUE2 system has been replaced by OnCore for new clinical trial submissions)

4.4. Add the details of each source of funding for this study.

Sponsor	Principal Investigator	Type of Funding
There are no items to display		

4.5. For those studies with a related award in the USC award system, Kuali Coeus, please use the "Find Now" button below to relate this study with the award(s):

Related Awards (uncheck checkbox to remove):

PI First Name	PI Last Name	Institutional Proposal Number	USC Award Number	Project Title	Prime Sponsor Name	Sponsor Name	Project Start Date	Co Investigators
Thomas	Buchanan	00019150		Southern California Clinical and Translational Science Institute		US-National Center for Advancing Translational Sciences	7/1/2015	Michael Hochman;

5. Type of Study Review

5.1. Select the type of review that you are requesting for this study:

○ Full Committee Review

● **Expedited Review**

○ Exempt Review

○ Coded Specimens/Data

5.2. **Attach the protocol here. For simple studies, a separate protocol may not be necessary. However, larger, complex, or multi-site studies require a fully developed protocol. If you have questions contact the IRB office to discuss.**

Name	Version	Modified
Lupus and fertility Twitter data study_v5_03.22.2019	0.06	3/22/2019 9:15 PM

For investigator-initiated trials at USC, a protocol template and protocol writing tips are available here: https://oprs.usc.edu/hsirb/biomedical/investigator-initiated-trials/

5.3. **Attach the sponsor's template informed consent here.**

Name	Version	Modified

There are no items to display

5.4. **If any study documents are password protected, enter the passwords here.**
N/A

5.5. **If there is a sponsor protocol number associated with this file, specify it here:**
N/A

6. Study Locations

6.1. **Select each campus the study will be associated with (check all that apply):**

☑ **HSC - Health Sciences Associated Locations**

☐ UPC - University Park Associated Locations

☐ CHLA

6.2. **Will any research covered by this application be conducted at any other site not affiliated with USC or CHLA?**
○ Yes ● **No**

6a. HSC Location(s)

This screen is required if you indicated HSC - Health Sciences Associated Locations (Question 6.1.)

6a.1. **Locations that recruitment, consent, and/or study procedures will be performed:** (check all that apply)

Location

- [ ] LAC+USC Medical Center
- [ ] LAC+USC Emergency Dept
- [ ] LAC+USC Outpatient Clinics
- [ ] LAC+USC 5P21 Building
- [ ] Keck Hospital of USC Facilities
- [ ] USC Norris Comprehensive Cancer Center Facilities
- [x] **Keck School of Medicine of USC**
- [ ] USC Eye Institute
- [ ] USC Healthcare Consultation Center I or II
- [ ] USC Center for Health Professions (CHP)
- [ ] USC School of Dentistry
- [ ] El Monte Comprehensive Health Center *
- [ ] H. Claude Hudson Comprehensive Center *
- [ ] Roybal Comprehensive Health Center *
- [ ] Verdugo Hills Hospital
- [ ] Other location (e.g., subjects home, community)

6a.2. Describe other location(s) at HSC:
N/A

6a.3. If you are conducting this research in an LAC+USC location, specify the room numbers:
N/A

6a.4. If you are conducting this research at a location marked with an asterisk "*", attach a letter of approval from the medical director.

Name	Version	Modified
There are no items to display		

9. Methods and Procedures - Selected Descriptors/Community Engaged Research

Note: The list of items below IS NOT an all-inclusive list of methods and procedures available to investigators. The list only includes items that will trigger additional questions specific to areas of research or are necessary for the review process.

9.1. This study will involve: (check all that apply)

- [ ] Prospective collection of data/specimens
- [x] Use of existing or retrospective data/specimens

9.6. Does your study involve community-engaged research (community-engaged research addresses community needs and involves the community in research plan, conduct of study, etc)?

○ Yes ● **No**

11. Research Objectives and Background

11.1. Describe the specific objectives or aims of the study and hypotheses or research questions. **(HSC: *refer to specific sections of the protocol/grant, if applicable*)**

The objective of this study is to conduct a content analysis of Twitter data published by users (in English) in the U.S. between 9/1/2017 and 10/31/2018 in order to examine attitudes toward reproductive health issues among patients with lupus. Please see the related section in the attached study protocol on page 4.

11.2. Provide a summary of the background of the study, and explain how this research will contribute to existing knowledge. Describe previous work that provides a basis to show that the proposed research can be carried out without undue risk to human subjects. Include relevant citations. **(HSC: *refer to specific sections of the protocol/grant, if applicable*)**

Our findings will shed light on whether Twitter provides a promising data source for garnering insights and attitudes about reproductive health issues expressed among lupus patients. The data will also help to determine whether Twitter might serve as a potential outreach platform for raising awareness of lupus and reproductive health and for implementing related health interventions. Please see the introduction in the attached study protocol, pages 1-4.

13. Methods and Procedures - Retrospective Studies/Existing Data

This screen is required if you indicated the use of existing/retrospective data or specimens (Question 9.1.)

13.1. Do the retrospective/existing data involve records/specimens from deceased individuals?

○ Yes ● **No**

13.2. Attach a copy of the Data Collection forms you intend to use. Data Collection forms include a summary of the variables to be recorded from the original source.

	Name	Version	Modified
	Transcript data collection form	0.02	3/5/2019 12:19 PM
	User timeline data collection form	0.02	3/5/2019 12:19 PM

13.3. Describe the method of collection for the records/specimens and how the data/specimens will be analyzed.

We will use data from the public network Twitter via Symplur Signals, a Web-based application for Twitter user data. After data cleaning (see page 6 in the study protocol) text classifiers (Table 2) will be used to identify topics in posts. Posts will be classified manually into the a-priori and emergent specific categories. Two independent team members will review the Twitter messages to code them based on the criterions listed in Table 2. Please note that any identifying and personal health information will be redacted from the dataset by the coders.

13.3.a. Specify the number of records/specimens you expect to use: **(*This is a deprecated field - only used for existing studies.*)**

13.4. Describe the method(s) by which subject records will be identified.
We will analyze the Twitter messages about lupus in the context of who sent them. The goal is to learn about patients' perspectives. Therefore, we will review but not collect nor store users' profile image, their Twitter handle, and profile description on Twitter. Since the �Tweet ID�, �Tweet URL�, �Profile thumbnail URL�, �Username� and �Display Name� in the dataset can potentially identify the person directly, we will remove these from the initial data collection sheet and use a unique code identifier instead. We will maintain the link between the unique code and the identifiable elements in a separate file. We will retain the data only for use in this project and destroy the identifiable (Tweet ID, Tweet URL, Profile thumbnail URL, Username and Display Name) information prior to the data analysis.

22. Special Subject Populations

22.1. Indicate any special subject populations you intend or expect to enroll in the research: (check all that apply)

- [ ] Normal Volunteers
- [ ] Employees or Students
- [ ] Adults not Competent to Consent (or likely to lose the capacity to consent during the study)
- [ ] Non-English Speaking Populations
- [ ] Minors (subjects under 18 years of age)
- [x] **Pregnant Women / Human Fetuses**
- [ ] Neonates (infants under 30 days old)
- [ ] Prisoners/Detainees
- [ ] Wards
- [ ] None of the above

24. Subject Recruitment and Informed Consent

24.3. Informed Consent and Waivers:

**** Please note that child assent and parental permission will be addressed on subsequent pages. Do not complete the following consent questions if adults will not be participating in the study. ****

Check the type(s) of consent or waiver of consent planned for this study: (check ALL that apply)

- [ ] Written/signed consent (participants will sign an informed consent document)

- [ ] An information sheet will be provided and/or verbal consent obtained

- [x] **Waiver of consent (participants will not be asked to sign a consent document or be given an information sheet)**

- [ ] Alteration of the elements of consent (participants will sign a consent document, but one or more of the basic required elements of consent will be altered or waived)

24.5. You indicated you are requesting a waiver of consent or a waiver/alteration of one or more elements of informed consent. The following questions are required:

24.5.1. The research involves no more than minimal risk to subjects and the waiver/alteration will not adversely affect the rights and welfare of the subjects because: (check ALL that apply and at least one answer from A at least one answer from B)

A. The study will: (check all that apply)

- [x] **Only collect retrospective data or be performing secondary data analyses on existing data**
- [ ] Only collect information from observation of public behavior
- [ ] Only collect information from standard of care procedures
- [x] **Not contact participants**
- [ ] Not include any sensitive information that could be considered harmful if known (HIV status, drug/alcohol treatment records, etc.)
- [x] **Other**

Please Explain:
We will analyze the Twitter messages about lupus in the context of who sent them. The goal is to learn about patients' perspectives. Therefore, we will review but not collect nor store users' profile image, their Twitter handle, and profile description on Twitter. Since the �Tweet ID�, �Tweet URL�, �Profile thumbnail URL�, �Username� and �Display Name� in the dataset can potentially identify the person directly, we will remove these from the initial data collection sheet and use a unique code identifier instead. We will maintain the link between the unique code and the identifiable elements in a separate file. We will retain the data only for use in this project and destroy the identifiable (Tweet ID, Tweet URL, Profile thumbnail URL, Username and Display Name) information prior to the data analysis. All analyses will adhere to the terms and conditions, terms of use, and privacy policies of Twitter.

B. All Data/Information collected will: (check ALL that apply)

- [ ] Not contain any identifiable information
- [x] **Be coded and the key codes kept separately and securely**
- [x] **Be kept in a locked/password protected area accessible only to study staff**
- [x] **Other**

Please Explain:
We will analyze the Twitter messages about lupus in the context of who sent them. The goal is to learn about patients' perspectives. Therefore, we will review but not collect nor store users' profile image, their Twitter handle, and profile description on Twitter. Since the �Tweet ID�, �Tweet URL�, �Profile thumbnail URL�, �Username� and �Display Name� in the dataset can potentially identify the person directly, we will remove these from the initial data collection sheet and use a unique code identifier instead. We will maintain the link between the unique code and the identifiable elements in a separate file. We will retain the data only for use in this project and destroy the identifiable (Tweet ID, Tweet URL, Profile thumbnail URL, Username and Display Name) information prior to the data analysis. All analyses will adhere to the terms and conditions, terms of use, and privacy policies of Twitter.

24.5.2. Explain why the research could not practicably be carried out without the waiver or alteration: (check ALL that apply)

- [ ] The data being collected are from existing records. Many of the subjects are lost to follow up, no longer seen at the hospital/facility, or deceased.
- [ ] Participation in this study does not involve personal contact. The participants are not available to provide informed consent.
- [x] **The study will be examining records from a large number of subjects. It is not feasible to attempt to contact all of them.**
- [ ] Other

24.5.3. Explain how, whenever appropriate, the subjects will be provided with additional pertinent information after participation: (check ALL that apply)

- [x] **There is no foreseeable need to provide information to the subjects. If there is a need, the IRB will be contacted to discuss the specific situation.**
- [ ] The study is observational and any results generated from the study will not be applicable to the subjects or the care of the subjects.
- [ ] Other

26. Participant Privacy and Data Confidentiality

26.1. Privacy Protections: Privacy is a participant's ability to control how other people see, touch, or obtain information about his/her self. Violations of privacy can involve circumstances such as being photographed or videotaped without consent, being asked personal questions in a public setting, being seen without clothing, being observed while conducting personal behavior, or disclosing information about abortions, HIV status, or illegal drug use.

Select the provisions to protect the privacy of the individual during screening, consenting, and conduct of the research: (check ALL that apply)

- [ ] Research procedures will be conducted in person in a private setting.
- [x] **Data will be captured and reviewed in a private setting.**
- [x] **Only authorized research study personnel will be present during research related activities.**

The collection of information about participants is limited to the amount necessary to

- [x] **achieve aims of the research.**
- [ ] Participants will not be approached in a setting or location that may constitute an invasion of privacy or could potentially stigmatize them.
- [x] **Other (specify below)**

26.1.1 Please specify:
We will analyze the Twitter messages about lupus in the context of who sent them. The goal is to learn about patients' perspectives. Therefore, we will review but not collect nor store users' profile image, their Twitter handle, and profile description on Twitter. Since the �Tweet ID�, �Tweet URL�, �Profile thumbnail URL�, �Username� and �Display Name� in the dataset can potentially identify the person directly, we will remove these from the initial data collection sheet and use a unique code identifier instead. We will maintain the link between the unique code and the identifiable elements in a separate file. We will retain the data only for use in this project and destroy the identifiable (Tweet ID, Tweet URL, Profile thumbnail URL, Username and Display Name) information prior to the data analysis. All analyses will adhere to the terms and conditions, terms of use, and privacy policies of Twitter.

26.2. Confidentiality Precautions: Confidentiality is an extension of the concept of privacy; it refers to the participant's understanding of, and agreement to, the ways identifiable information will be collected, stored, and shared. Identifiable information can be printed information, electronic information, or visual information such as photographs.

How will the research data/specimens be labeled? (check ALL that apply)

- [ ] Data and/or specimens will be directly labeled with personal identifying information. (Identifiable)
- [x] **Data and/or specimens will be labeled with a code that the research team can link to personal identifying information. (Coded)**
- [ ] Data and/or specimens will not be labeled with any personal identifying information, nor with a code that the research team can link to personal identifying information. (Anonymous)
- [x] **Other (explain below)**

26.2.1. Please explain how data will be recorded:
Will remove identifiable information from the data collection sheet, maintain the link between sheets in a separate file, destroy the identifiable information prior to the data analysis.

26.2.2. If you are recording data in more than one way, please explain and provide justification:
We will analyze the Twitter messages about lupus in the context of who sent them. The goal is to learn about patients' perspectives. Therefore, we will review but not collect nor store users' profile image, their Twitter handle, and profile description on Twitter. Since the �Tweet ID�, �Tweet URL�, �Profile thumbnail URL�, �Username� and �Display Name� in the dataset can potentially identify the person directly, we will remove these from the initial data collection sheet and use a unique code identifier instead. We will maintain the link between the unique code and the identifiable elements in a separate file. We will retain the data only for use in this project and destroy the identifiable (Tweet ID, Tweet URL, Profile thumbnail URL, Username and Display Name) information prior to the data analysis. All analyses will adhere to the terms and conditions, terms of use, and privacy policies of Twitter.

26.3. Study data/specimen will be stored:

- [ ] Physically
- [x] **Electronically**

Which devices will have study data:

- [x] **Local computers/laptops**
- [ ] Removable drives (USB, external drives)
- [x] **Local Server(s)**
- [ ] External Servers (including cloud based services)

Please confirm that, at a minimum, the following measures will be taken and enforced:

- [x] **Electronic data will be stored with appropriate electronic safeguards, such as unique usernames/passwords, and limited to authorized study personnel. Dual factor authentication will be used, if feasible.**
- [x] **Security software (firewall, antivirus, anti-intrusion) will be installed and regularly updated in all servers, workstations, laptops, and other devices used in the study**
- [x] **All computers with access to study data will be scanned regularly (for viruses and spyware, etc.) and problems will be resolved**

Other Measures:

- [x] **Locked office**
- [x] **Restricted access to authorized study personnel**
- [x] **Secure computer/laptop**
- [x] **Encryption of digital data**
- [x] **Security software (firewall, antivirus, anti-intrusion) is installed and regularly updated in all servers, workstations, laptops, and other devices used in the study**
- [ ] Audio and/or video recordings will be transcribed and then will be destroyed
- [ ] Audio and/or video recordings will be modified to eliminate the possibility that study participants could be identified
- [ ] Photos or images will be modified to eliminate the possibility that study participants could be identified

**Note: Data stored on a cloud service must comply with USC policy

26.4. Will identified data and/or specimens be released to a third party (such as a study sponsor, federal agency, or another institution)?

○ Yes ● **No**

26.5. What will happen to the research data and/or specimens at the conclusion of the study?

(check ALL that apply)

- [ ] Direct identifiers and/or the key to the codes will be destroyed upon completion of the research (all data/specimens will be stripped of identifying information and/or the key to codes destroyed, paper documents shredded, electronic files purged, electronic media securely erased)
- [ ] Retained for study record keeping purposes per institutional policy
- [ ] Retained by the investigator for future research use
- [ ] Retained for future research use (create data or tissue repository/bank)
- [ ] Restricted use data will be destroyed or returned to the source
- [ ] No direct or indirect identifiers are being collected. The anonymous data and/or specimens will be retained at the discretion of the investigator
- [ ] This research is a clinical trial conducted under FDA regulations. Direct identifiers and/or the key to the codes will be destroyed as directed by the sponsor (IND/IDE holder) in accordance with FDA regulations
- [ ] The NIH requires that the records be retained for three years following the completion of the study
- [x] **Other (specify below)**

26.5.1 Please specify:
Since the �Tweet ID�, �Tweet URL�, �Profile thumbnail URL�, �Username� and �Display Name� in the dataset can potentially identify the person directly, we will remove these from the initial data collection sheet and use a unique code identifier instead. We will maintain the link between the unique code and the identifiable elements in a separate file. We will retain the data only for use in this project and destroy the identifiable (Tweet ID, Tweet URL, Profile thumbnail URL, Username and Display Name) information prior to the data analysis.

26.6. Do you have, or plan to apply for, a Certificate of Confidentiality for this study?

NOTE: All NIH-funded research that meets the definition of human subjects research (including exempt research in which subjects can be identified), is collecting or using human biospecimens that are identifiable or that have a risk of being identifiable, involves the generation of individual level human genomic data, OR involves any other information that might identify a person is automatically issued a Certificate of Confidentiality by NIH.

○ Yes ● **No**

35. Is the HIPAA Privacy Rule Applicable?

35.1. Do you intend to access, review, collect, use or disclose protected health information (PHI) in your research? Answer yes if you intend to do any of the following:

- Look at medical records (paper or electronic) to identify potential research participants
- Look at clinic logs to identify potential research participants
- Record demographic information obtained from medical records (paper or electronic)
- Record health information obtained from medical records (paper or electronic)
- Obtain information from laboratory reports, pathology reports, radiology reports or images,

or other reports from medical or mental health testing and treatment
- Obtain information from medical billing records
- Record or use medical record numbers or other information that could be used to identify an individual (review the list of HIPAA identifiers below)
    - Name/Initials
    - Street address, city*, county*, precinct*, zip code*, or equivalent geocodes*
    - All elements of dates (except year) directly related to an individual (date of birth, admission date, discharge date, date of death)*
    - Elements of date, including year, for persons 90 or older
    - Telephone number
    - Fax number
    - Electronic mail address
    - Social Security Number
    - Medical record number
    - Health plan identification number
    - Account number
    - Certificate/license number
    - Vehicle identifiers and serial numbers, including license plate number
    - Device identifiers and serial number
    - Web addresses (URLs); Internet IP addresses
    - Biometric identifiers, including finger and voice print
    - Full face photographic images and any comparable images
    - Any other unique identifying number, characteristic, or code*

○ Yes ● **No**

39. Conflict of Interest Information

39.1. Indicate the Study team member(s) that have a potential conflict of interest. For each person to be designated, click on his/her name and select the disclosure(s) that should be associated with this study.

Study Staff	Role	Conflicts
Katja Reuter	Principal Investigator	No conflicts identified

40. Additional Supporting Documents

40.1. Attach any other documents that have not been specifically requested in previous questions, but are needed for IRB review.

Name	Version	Modified
There are no items to display		

40.2. If there is any additional information that you wish to communicate about the study include it below. Please note, this section should not be used instead of the standard application items.
N/A

99. Instructions for Submission

You have reached the end of the application. When you are sure of the content, the following steps may be taken to submit your application for review.

1. Click the **"Finish"** button on the top or bottom application navigator bar to return to the workspace.
2. Use the **Hide/Show Errors** above to determine that all sections of the application are filled out correctly.
3. Use the **"Send Study Ready Notification"** activity to send an email to the Principal Investigator and Co-Investigators with instructions for reviewing and submitting the application.
4. **All listed Co-Investigators (indicated in item 2.1.) must use the "Agree to Participate" activity and answer yes.**
5. Once all the Co-Investigators have agreed to participate, the **Principal Investigator** (indicated in item 2.1.) can submit the application by using the **"Submit Application to ____"**, where ____ indicates the IRB you are submitting to.
6. The PI will have to check the PI endorsement box. The PI will also have to check the student endorsement box if it is applicable.
7. The application is submitted. The state indicator in the top left of the workspace will no longer display Pre Submission.
8. The PI and Study Contact Person will receive an email confirming the application has been submitted.
