# Supplementary figures and images for: Insights From Twitter Conversations on Lupus and Reproductive Health: Protocol for a Content Analysis
Source: JMIR Res Protoc. 2020 Aug 26;9(8):e15623. doi: 10.2196/15623 (PMC7481870; doi:10.2196/15623)

**Multimedia Appendix 5. Data extraction and cleaning flow diagram.**

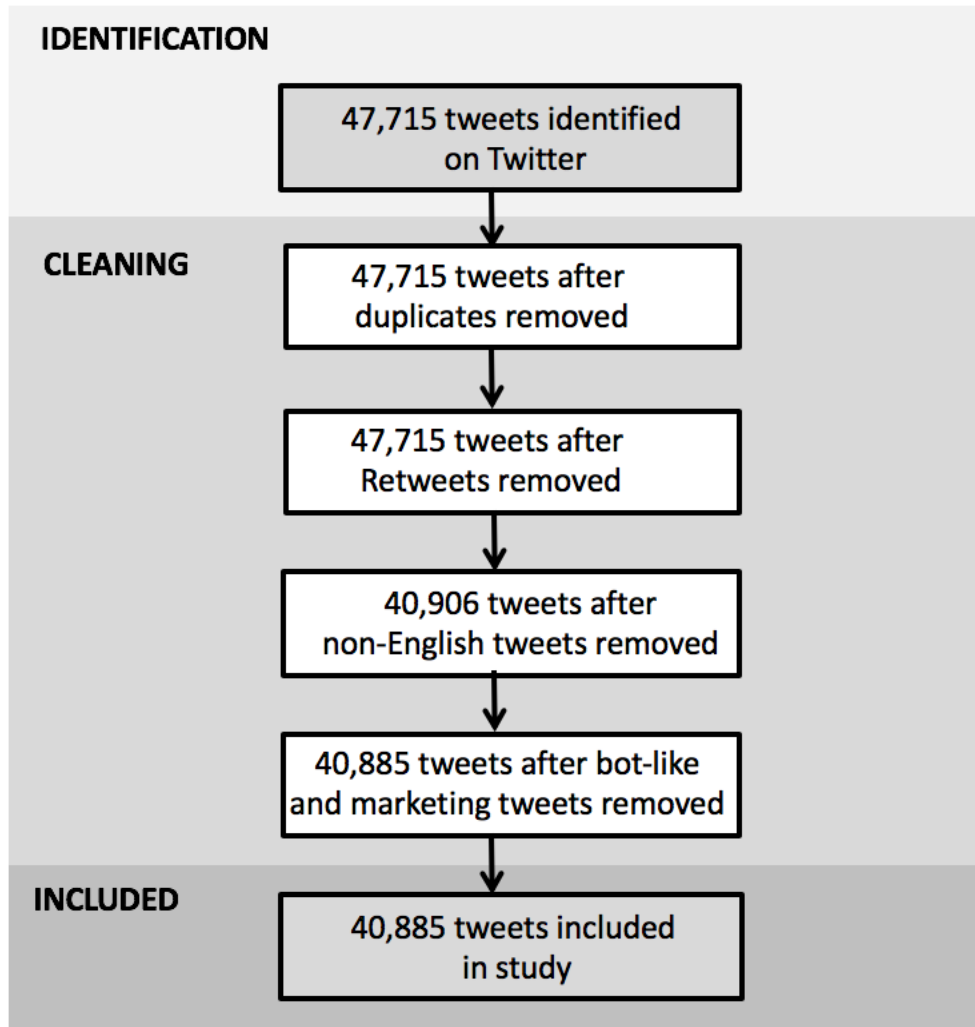

Supplement: Multimedia Appendix 5 [file resprot_v9i8e15623_app5.pdf]
